# Atypical Legionnaires' Disease in the Setting of Suspected Recurrent Lung Cancer

**DOI:** 10.7759/cureus.24760

**Published:** 2022-05-05

**Authors:** Merna Haridi, Alana Hutcheson, Beatriz De Faria, Mohamed Saleh

**Affiliations:** 1 Family Medicine, St. Martinus University, Willemstad, CUW; 2 Family Medicine, Nova Southeastern University Dr. Kiran C. Patel College of Osteopathic Medicine, Fort Lauderdale, USA; 3 Family Medicine, Pontiac General Hospital, Pontiac, USA

**Keywords:** immunocompromised patients, infection prevention and control, lung cancer, legionnaires disease, legionella infection

## Abstract

Legionnaires’ disease is a type of pneumonia caused by *Legionella* bacteria. This type of bacteria can be found anywhere across the world, mostly in moist environments (e.g., ground soil, rivers, lakes). More importantly, *Legionella* can multiply in water systems such as air conditioners, which is a common source of outbreaks nationwide, particularly during the summer months. We present a unique clinical course of Legionnaires’ disease with suspected underlying recurrent lung cancer in a 77-year-old man during an outbreak that originated in a small city near our hospital. The patient presented to Urgent Care and after initial assessment, was admitted to the Internal Medicine Unit. He underwent supportive treatment with antibiotic therapy and oxygen, and was discharged one week after admission with improvement. The patient returned to Urgent Care a few weeks later with worsening dyspnea, where he was then transferred to another hospital for admission to the Intensive Care Unit (ICU), and later died. We report this special case to bring awareness to physicians of the possibility and importance of early detection and prompt management of Legionnaires’ disease in lung cancer and critically ill patients with possible environmental risk factors. Prompt detection and management of L*egionella pneumophila* allows for a greater chance of a favorable prognosis, particularly in the immunocompromised.

## Introduction

The incidence of Legionnaires’ disease is about 1.4-1.8 cases in 100,000 people [1]. Most cases are due to *Legionella pneumophila*, an aerobic, gram-negative, intracellular bacteria. *Legionella* is typically transmitted via inhalation of aerosols from contaminated water sources or soil [2]. Typically, it presents in the summer months between July and September and can present as an outbreak or as sporadic cases [3]. Per the Michigan Department of Health and Human Services, there was an increase in cases of Legionnaires’ disease in July 2021 in multiple counties across Michigan [4]. Legionnaires’ disease typically presents as pneumonia with fever, cough, and shortness of breath along with diarrhea and vomiting. Rust-colored sputum or confusion may also be present [3]. We bring forward a sporadic case that was presented in early fall with suspected underlying lung cancer to highlight the connection between critically ill patients, especially those with lung cancer, and the possibility of developing Legionnaires’ disease.

## Case presentation

A 77-year-old man who was an active smoker with a 30 pack-year smoking history and past medical history of hypertension, chronic obstructive pulmonary disease (COPD), and lung cancer with lobectomy presented to the urgent care with a one-day history of productive cough and dyspnea. He had noted progressively worsening cough, shortness of breath on exertion, and diarrhea. He denied any nausea, vomiting, chest pain, abdominal pain, or weight changes. He denied any sick contacts and did not travel anywhere recently.

Upon arrival at the hospital, he was noted to have a temperature of 101.7℉, heart rate of 81 bpm, blood pressure 146/56, respiratory rate of 16, and O2 saturation was 93. Further physical examination revealed a moderately distressed male due to dyspnea. A pulmonary examination revealed bilateral crackles. He was noted to have normal heart sounds without any murmurs, rubs, or gallops. The abdominal examination was normal. He did not have rashes or peripheral edema. His respiratory distress was initially treated with non-invasive ventilatory support. His laboratory test results on admission are shown in Table 1.

**Table 1:** Laboratory test results ref: reference range

Tests	Results (reference range)
Sodium	129 Lmmol/L (ref, 136-145)
Potassium	4.1 mmol/L (ref, 3.5-5.1)
Creatinine	1.5 Hmg/dL (ref, .8-1.3)
Glomerular filtration rate	48 mL/min
WBC	10.5 K/uL (ref, 3.4-10.5)
Neutrophils	3.0%
Lymphocytes	12.0%
Hemoglobin	11.6 Lg/dL (ref, 11.7-16.0)
Platelets	230 K/uL (ref, 120-400)

**Figure 1:**
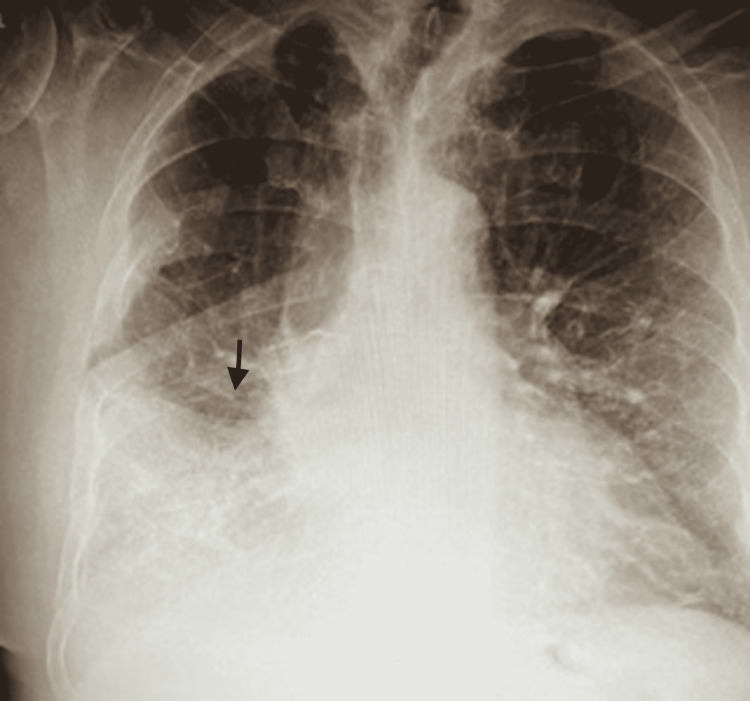
Chest x-ray demonstrating right lung opacity Arrow indicates the infiltrate

He was treated with intravenous Lasix 20 milligrams, ceftriaxone 2 grams, and fluids for suspected pneumococcal community-acquired pneumonia (CAP) in urgent care, and was admitted to the hospital. Chest radiograph (CXR) done on the same day showed interval development of consolidation involving the right lower lobe since prior examination (Figure 1). Upon admission, treatment was continued with 2 grams of intravenous ceftriaxone every 24 hours and 100 milligrams of doxycycline every 12 hours. His oxygen requirement remained high without any improvement. Due to concerns for CAP, blood cultures via peripheral blood draw and urinary antigens for *Mycoplasma pneumoniae* and *Streptococcus pneumoniae* were obtained. 

**Figure 2:**
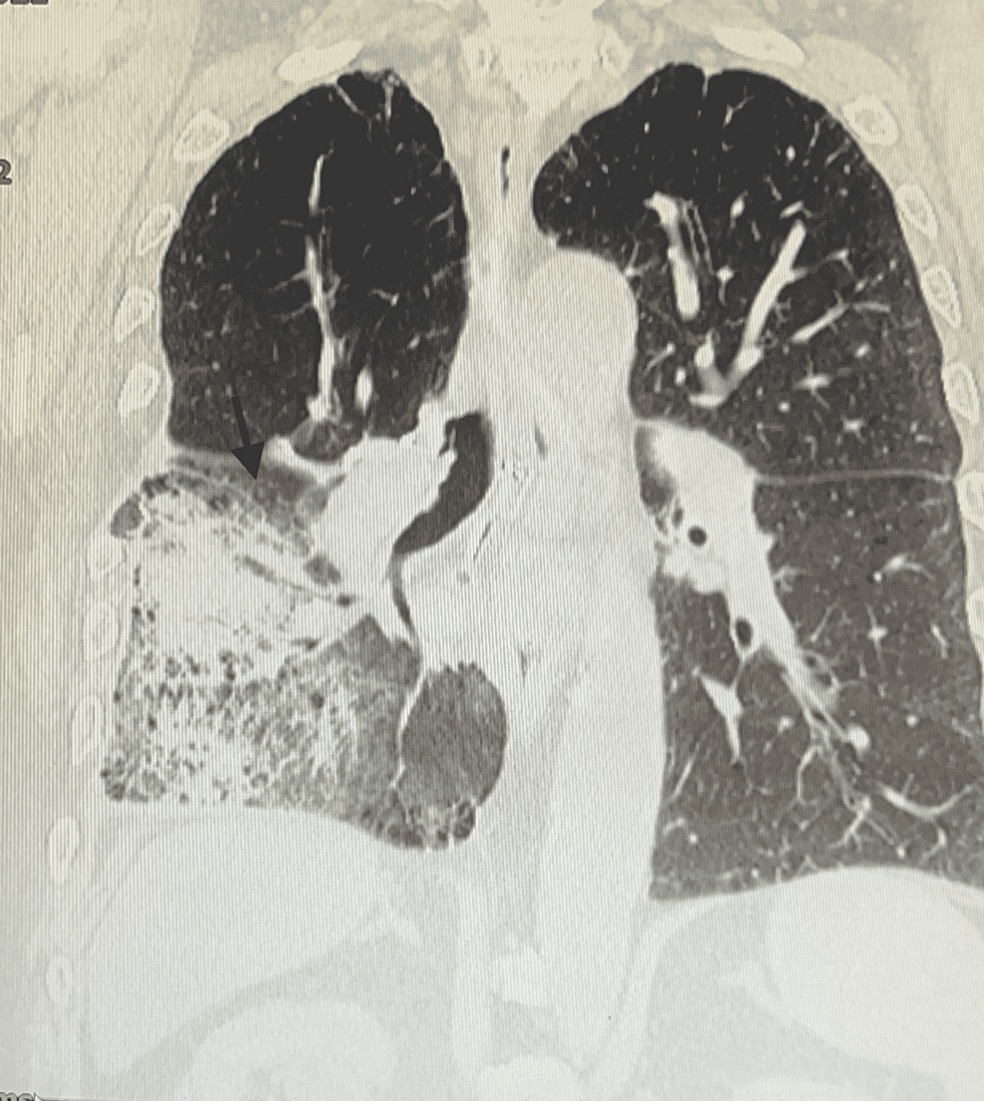
Chest CT demonstrating right lung opacity and infiltrating mass Arrow indicates the infiltrate

ECG revealed sinus arrhythmia with incomplete right bundle branch block (RBBB) and T-wave inversions. After day one of admission, a computed tomography (CT) scan showed prominent infiltrate in the right mid and lower lung suggesting a large mass (Figure 2). On day two, he spiked a fever of 103.6℉ with increased production of orange-like sputum. *Legionella* urine antigen was positive for *L. pneumophila* and the antibiotics were discontinued. The infectious diseases team was consulted.

He was started on intravenous azithromycin 500 milligrams every 24 hours, levofloxacin 500 milligrams for five days, and ceftriaxone 1 gram. ECG showed prolonged QT interval. On day three, his condition improved dramatically; GI symptoms ceased and the fever subsided. ECG was repeated and showed sinus rhythm with prolonged QT interval and T-wave inversions. On day six, his serum sodium level increased to 135 mEq/L, and his chloride normalized to 98 mEq/L. Electrolytes and renal function improved. The patient’s clinical status improved, and he was discharged home on 3L of oxygen and oral levofloxacin 500 milligrams after a six-day hospital stay.

Three weeks following the discharge of the patient, he returned to Urgent Care with dyspnea and elevated cardiac troponins. He was immediately transferred to the Cardiology Unit in another hospital and was intubated. He died a few days after hospitalization. No autopsy was performed.

## Discussion

*Legionella* is an intracellular, gram-negative bacteria that is commonly known to cause nosocomial pneumonia. *Legionella *infection can present as a febrile illness, pneumonia, GI symptoms (nausea, diarrhea), transaminitis, and hyponatremia. We report a case of *L.* *pneumophila* pneumonia in a patient with suspected nodular lung cancer. This case demonstrates the prompt need to test for and treat *Legionella* in immunosuppressed patients once it is suspected, despite the presence or absence of environmental factors.

CAP is defined as the presence of an infiltrate on the CXR, in addition to at least one of the following signs and symptoms: dyspnea, cough, sputum production, and abnormal breath sounds [5]. On the other hand, *Legionella* CAP includes the mentioned signs and symptoms, in addition to at least one positive microbiological test for the organism. Identifying *Legionella* in patients presenting with CAP has proven to be challenging over time [5]. However, several studies have proposed a clinical rule that can be used to quickly identify it [6]. This rule is known as the CAP incidence study (CBPIS), which has a system of 17 points based on the evaluation of serum creatinine, sodium and lactate dehydrogenase (LDH), temperature, headache, smoking, and vomiting [6]. Although this proposal has proven to have low sensitivity and/or specificity, a scale with the different criteria in *Legionella* pneumonia is needed for future practice.

*Legionella* species cause a severe form of CAP as well as a high mortality rate of about 10% [7]. More importantly, the mortality rate in patients with *Legionella* may be as high as 27% in patients without adequate antibiotics management on admission [7]. Early identification of *Legionella* is of utmost importance in patients presenting to the Emergency Department or Urgent Care with respiratory symptoms. Research has shown that a delay in the appropriate therapy for *L. pneumophila* pneumonia is associated with an increased rate of mortality [8]. A urinary antigen test has shown 100% specificity in detecting *L. pneumophila* and, thus, should be considered in suspected cases. Physicians should consider testing for this organism and administer the appropriate anti-*Legionella* antibiotics promptly to the patients with risk factors, more importantly, the critically ill.

This patient presented to our hospital with a one-day history of productive cough and dyspnea. He has a medical history significant for multiple lung pathologies, which are risk factors for developing Legionnaires' disease [8]. He also had a suspected recurrence of his lung cancer, making him immunocompromised. We hypothesized that our patient’s underlying lung pathologies made him more susceptible to developing severe disease and put him at risk of a higher mortality rate. No autopsy was performed on the case under review. However, prompt diagnosis and treatment may improve the prognosis of Legionnaires' disease in immunocompromised patients.

## Conclusions

Legionnaires’ disease is caused by a gram-negative intracellular bacteria known as *Legionella*. It has been shown to rarely cause CAP but can be associated with mortality, especially in immunosuppressed individuals. We report a case of a 77-year-old man with Legionnaires’ disease and underlying suspected lung malignancy. The main goal of this case report is to facilitate early recognition and treatment of *Legionella* in immunosuppressed patients with lung cancer who present with pulmonary symptoms. This approach will lower the chances of mortality earlier in the disease course.
